# Identification of Bunyamwera and Possible Other *Orthobunyavirus* Infections and Disease in Cattle during a Rift Valley Fever Outbreak in Rwanda in 2018

**DOI:** 10.4269/ajtmh.19-0596

**Published:** 2020-04-20

**Authors:** Marie Fausta Dutuze, Angelique Ingabire, Isidore Gafarasi, Solange Uwituze, Manassé Nzayirambaho, Rebecca C. Christofferson

**Affiliations:** 1 School of Veterinary Medicine, Louisiana State University, Baton Rouge, Louisiana;; 2 College of Agriculture and Animal Sciences and Veterinary Medicine, University of Rwanda, Kigali, Rwanda;; 3 Rwanda Agriculture Board, Kigali, Rwanda;; 4 School of Public Health, University of Rwanda, Kigali, Rwanda

## Abstract

In 2018, a large outbreak of Rift Valley fever (RVF)–like illness in cattle in Rwanda and surrounding countries was reported. From this outbreak, sera samples from 157 cows and 28 goats suspected to be cases of RVF were tested to confirm or determine the etiology of the disease. Specifically, the hypothesis that orthobunyaviruses—Bunyamwera virus (BUNV), Batai virus (BATV), and Ngari virus (NRIV)—were co-circulating and contributed to RVF-like disease was tested. Using reverse transcriptase-polymerase chain reaction (RT-PCR), RVFV RNA was detected in approximately 30% of acutely ill animals, but in all cases of hemorrhagic disease. Seven cows with experienced abortion had positive amplification and visualization by gel electrophoresis of all three segments of either BUNV or BATV, and three of these were suggested to be coinfected with BUNV and BATV. On sequencing, five of these seven cows were conclusively positive for BUNV. However, in several other animals, sequencing was successful for some but not all segments of targeted viruses BUNV and BATV. In addition, there was evidence of RVFV–orthobunyavirus coinfection, through RT-PCR/gel electrophoresis and subsequent Sanger sequencing. In no cases were we able to definitely identify the specific coinfecting viral species. This is the first time evidence for orthobunyavirus circulation has been molecularly confirmed in Rwanda. Furthermore, RT-PCR results suggest that BUNV and BATV may coinfect cattle and that RVFV-infected animals may be coinfected with other orthobunyaviruses. Finally, we confirm that BUNV and, perhaps, other orthobunyaviruses were co-circulating with RVFV and contributed to the burden of disease attributed to RVFV in Rwanda.

## INTRODUCTION

The group of viruses commonly referred to as bunyaviruses have caused diseases of zoonotic and economic importance globally since the beginning of the twentieth century.^1–4^ In Africa, Rift Valley fever virus (RVFV) was first identified and characterized in 1912 and further detected as the etiological agent of an epizootic outbreak in Kenya in 1930.^5–7^ Rift Valley fever virus is a vector-borne virus that causes disease in humans, livestock, and wildlife ruminant species.^1,6,8,9^ In animals, it is clinically characterized by abortions and stillbirths, hepatitis, and hemorrhagic fever in severe cases.^8,10,11^ In humans, the mild form is characterized by a self-limiting febrile illness, but individuals may progress to severe disease manifesting as hemorrhagic fever, encephalitis, vision loss, and conjunctivitis.^8,12–18^ These clinical manifestations are common in many diseases caused by bunyaviruses.^2,19,20^

Although RVFV has a broad range of identified vectors consisting of mosquitoes, ticks, and flies, the primary vectors are *Aedes* spp. and *Culex* spp. mosquitoes.^21–28^ Rift Valley fever virus consistently circulates in domestic ruminants and human populations throughout different countries of Africa and the Arabian Peninsula where it causes significant outbreaks associated with livestock mortality, associated economic losses, and human mortalities.^1,7,29–34^ The virus is maintained during interepidemic periods by transovarial transmission in *Aedes* mosquitoes, and the resurgence of outbreaks is facilitated by periodic rainfall and flooding due to the intertropical convergence of air currents from southern and northern hemispheres which leads to a seasonal emergence of infected *Aedes* mosquitoes.^27,35^ After the emergence of infected *Aedes* mosquitoes, an outbreak may be sparked, but the epidemic is likely maintained by *Culex* spp*.*^35^ In East Africa, this cycling pattern has been recognized since the 1970s with large outbreaks occurring in 1977 (Egypt),^15^ 1997–1998 (Kenya),^33^ 2006–2008 (Somalia, Tanzania, and Kenya),^36,37^ and 2018 (Kenya, Uganda, and Rwanda).^37–39^

In Rwanda, the only bunyavirus regularly surveilled is RVFV and is often clinically diagnosed. The first molecularly confirmed case of RVF was reported in Bugesera in 2011 after unusually high rates of abortions in domestic ruminants (mainly cattle) during the period of May–June were observed.^40,41^ Since then, RVF has become endemic with sporadic cases reported year round in the eastern part of the country and higher intensity outbreaks in May–June and December–January following the rainy seasons of March–April and October–November.^40,41^ For prevention, livestock are recommended to be regularly vaccinated with live attenuated Rift Valley fever vaccine prepared from Smithburn’s attenuated strain of RVFV (RIFTVAX, Kenya Veterinary Vaccines Production Institute, Nairobi, Kenya).^40^ However, vaccine uptake and compliance rates are unknown.

Although RVFV is the most recognized and significant arbovirus in the region, many other zoonotic Bunyaviruses, such as Bunyamwera virus (BUNV), Batai virus (BATV), and Ngari virus (NRIV), circulate in East Africa (reviewed in ref. 3). Bunyamwera virus was first identified in Uganda (1943) and since has been regularly isolated in Tanzania, Kenya, and Uganda between 1945 and 2012.^3,42–44^ Batai virus was first identified in Uganda (1966) and highly suspected in Sudan by serological assay (1988).^45,46^ Ngari virus was first identified as an etiological agent of fatal hemorrhagic fever outbreak in Kenya (1997–1998).^43,47,48^ Despite their obvious potential to be of public and One Health importance, BUNV, BATV, and NRIV are not ordinarily included in diagnostic panels and could be cryptically circulating throughout sub-Saharan Africa.

Bunyamwera virus, BATV, and NRIV belong to the taxonomic genus *Orthobunyavirus*, and NRIV is the natural reassortant of BUNV and BATV (BUNV_L_BATV_M_BUNV_S_).^48^ Bunyamwera virus, BATV, and NRIV are transmitted by similar vectors as RVFV and infect similar vertebrate hosts, and infection with these viruses have been characterized with similar clinical manifestations.^47,48^ Specifically, the disease associated with BUNV has been reported to cause mild symptoms, such as fever, joint pain, and rash, in many mammals, including humans.^42,49–51^ Batai virus causes a mild flu-like illness in humans and is associated with a more severe disease in ruminants where it is manifested by abortions, premature births, and genetic defects.^49,52,53^ Ngari virus has been associated with fatal hemorrhagic fevers in both humans and ruminants.^54,55^ In 2018, the ICTV suggested a reorganization of Bunyavirus taxonomy, whereby the Rift Valley fever virus is now classified in the family Phenuiviridae and the orthobunyaviruses are now in the family Peribunyaviridae. However, this reorganization has not been uniformly adopted.^56,57^

In 2018, there were unusually heavy rainfalls that led to explosion of RVFV in East African countries (Rwanda, Kenya, Uganda, and Tanzania). This was the most intense occurrence of RVF in Rwanda as it caused many cases of death and/or abortion cases in the ruminant population and the deaths of two veterinarians.^38,39^ In addition, there was likely an underreporting of RVF cases of small ruminants because of their low economic and cultural importance in Rwanda. Diagnosis of cattle was primarily by clinical manifestations, not by molecular techniques.

The outbreak started in Ngoma and Kirehe districts (Eastern Province) of Rwanda at the end of May, and there were still many cases reported through the end of July in these two districts. The outbreak spread to other parts of the other districts of Eastern Province (Kayonza, Gatsibo, and Rwamagana). The Southern Province was also heavily affected followed by the Northern Province. The Western Province and Kigali city were affected late and at low levels. Animal movements were banned from mid-June to the end of July in an effort to slow the progress of the outbreak. Widespread human cases were not documented, excepting the deaths of two veterinary officers. The Rwanda Agriculture Board initiated a massive vaccination campaign of health and nonpregnant animals, and transmission was interrupted and the outbreak ended in July 2018.

Some samples from abortions were tested to confirm RVFV early in the outbreak, but diagnosis was mostly made based on clinical signs. Thus, we undertook to molecularly confirm RVFV as the etiological agent of RVF-like illness, and further tested samples to determine whether BUNV, BATV, or NRIV might be co-circulating and contributed to the burden of disease.^3^

## MATERIALS AND METHODS

### Ethical considerations.

This study was performed in conjunction with the Rwanda Agriculture Board and approved by the Institutional Review Board of the University of Rwanda in the College of Medicine and Health Sciences (Ref 026/17 DRIPGS/2017 of October 19, 2017) and by the Louisiana State University Institutional Animal Care and Use Committee (protocol #17-085).

### Sample collection and handling.

One hundred fifty-seven blood samples were collected from cattle and 28 from goats suspected of RVFV infection in Rwanda between May 29 and July 25, 2018. The inclusion criteria for blood collection were at least one of the following: having aborted in less than a week, presenting with signs of hemorrhagic fever, history of death suspected for RVF in the same farm in less than 3 days, and history of abortion suspected to be associated with RVF in less than 3 days. Calves whose mothers presented with signs of hemorrhagic fever or had died of suspected RVF within 3 days were sampled as well. Blood was collected in 5-mL purple-top vacutainer tubes (without anticoagulant). Demographic information of farmers such as address and complete identification of sampled animals (age, gender, breed, clinical signs, status of RVFV vaccination, and breed) were also collected. From the field, samples were transported to the virology laboratory of Rwanda Agriculture Board immediately at Rubirizi station. The samples were then put into 2-mL Eppendorf tubes and kept in −80°C, and subsequently, the samples were shipped to Louisiana State University where they were tested for RVFV and BUNV, BATV, and NRIV as described in the following paragraphs.

### Viral RNA extraction and cDNA synthesis.

Each sample was stored in DNA/RNA Shield (Zymo Research, #R1100, Irvine, CA). Before extraction of RNA, TRI Reagent (Sigma-Aldrich, #T9424, St. Louis, MO) was added and then viral RNA was extracted using Direct-zol^TM^ MiniPrep kit (Zymo Research, #R2052) according to the manufacturer’s instructions. First-step total cDNA synthesis was performed on viral RNA using random hexamers and 1 μL dNTPs and run on a thermocycler at 65°C for 5 minutes. Ten microliters of sample was then mixed with 2 μL 10× RT buffer, 4 μL 25 mM MgCl_2_, 2 µL 0.1 m dithiothreitol (DTT), 1 µL RNase OUT, and 1 µL SuperScript III RT kit (Invitrogen, #18080-051, Carlsbad, CA). The samples were then run on the thermocycler with the following cycling parameters: 25°C for 10 minutes, 50°C for 50 minutes, and 80°C for 5 minutes. One microliter µL of RNase H was added, and the samples were put back on the thermocycler at 37°C for 20 minutes. Totally, 21 µL per sample was collected and kept in −20°C until use.

### Primer design for BUNV, BATV, and NRIV.

The success of primers was confirmed using viral stocks: 6547-8, MM2222, and DAK-AR D28542 as the strain designations for BUNV, BATV, and NRIV, respectively, that were used throughout this study (Supplemental Figure S1). The prototype strain of BUNV isolated from *Aedes* spp. mosquitoes in Uganda in 1943 is 6547-8 (GenBank accession numbers: X14383, M11852, and X73465). The prototype of BATV isolated from *Culex* spp*.* mosquitoes in Malaysia in 1955 is MM2222 (GenBank accession numbers: AB257766, JX8446595-97, and X73464). DAK-AR D28542 strain of NRIV was isolated from *Aedes* male mosquitoes in Senegal in 1985 (GenBank accession number: AY593728-29). The viruses were obtained from the World Reference Center for Emerging Viruses and Arboviruses at the University of Texas Medical Branch (UTMB).

PCR primers were designed using IDTDNA (https://www.idtdna.com) PrimerQuest tool for all three gene segments for each virus (Supplemental Table S1): small (S), medium (M), and large (L) segments. The segments were tested for cross-reactivity (Supplemental Figure S1). As expected, the BUNV S and L primers cross-amplified the NRIV S and L segments, and the NRIV L primers cross-amplified the BUNV L segment. However, the NRIV S primers only weakly cross-amplified the BUNV S segment, and there was no cross-amplification of the BUNV M segment by the NRIV M primers, nor was there cross-amplification of NRIV M segment by the BATV primers or vice versa (Supplemental Figure S1). Amplification of targets was performed on the thermocycler with the following cycling parameters: 95°C for 15 minutes; 35 cycles of 94°C for 30 seconds, 54°C for 30 seconds, 72°C for 1 minute, and 72°C for 10 minutes; and a 10°C hold until samples were removed and placed in −20°C until further use. The primer sequences, size of amplicon, and accession number of gene segments are given in Supplemental Table S1.

### Targeted cDNA amplification and sequencing.

To test the aforementioned primers for sequencing abilities, we sequenced cDNA from stocks of BUNV, BATV, and NRIV from the WHO reference center (strains 6547-8, MM2222, and DAK-AR D28542, respectively). Sequencing was performed by Gene Probes and Expression Systems Laboratory of the Division of Biotechnology and Molecular Medicine at the Louisiana State University School of Veterinary Medicine. Sequencing was performed on a Beckman Coulter 8800 (Pasadena, CA) using the manufacturer's reagents and methods.^58^ All amplicons were successfully sequenced, and when blasted using NCBI Nucleotide BLAST (https://blast.ncbi.nlm.nih.gov/Blast.cgi), all segments identified as belonging to their respective viruses (Supplemental Table S2).

Samples were first tested for RVFV infection by conventional PCR using primers targeting the conserved 90-nt region of the L segment and using the same cycling parameters as in ref.^59^ Amplicons were stained by GelRed (Biotum, Cat No. 41003), run on 2% agarose gel with 1× TAE buffer at 100 V for 1 hour, and visualized with Bio View UV Light transilluminator. cDNA of RVFV-negative samples were pooled by 5–7 according to the districts of origin and were tested for BUNV, BATV, and NRIV. Samples belonging to positive pools were then tested individually. Rift Valley fever virus–positive samples were also tested for the three orthobunyaviruses. Detection of the three orthobunyaviruses was done by targeting all the three segments S, M, and L for each virus by PCR.

Positive samples via RT-PCR were then sent to the Gene Probes and Expression Systems Laboratory of the Division of Biotechnology and Molecular Medicine at the Louisiana State University School of Veterinary Medicine for sequencing. Sequencing was performed on a Beckman Coulter 8800 (Pasadena, CA) using the manufacturer’s reagents and methods.^58^ For all but one sample, sequences generated from the forward and reverse primers were aligned using NCBI Nucleotide BLAST (https://blast.ncbi.nlm.nih.gov/Blast.cgi), and the aligned FASTA files were then used to determine the identity of the sequences using BLASTnt (for one positive sample (Batai, 109S), double coverage was not achieved before sample volume was depleted and only the forward primer–derived sequence is available). All sequences were submitted to the GenBank using the BankIt submission tool. We classify RT-PCR positive, sequencing negative as “suggestive” and RT-PCR, sequencing positive samples as “positive.”

## RESULTS

### Rift Valley fever diagnosis confirmation and sample demographics.

There were more cows sampled than goats because of the economic and cultural importance of cows. Twelve goats were positive to RVFV, whereas 44 cattle were positive to RVFV via RT-PCR. More than 97% of samples were female, with the exception of four male calves included because their mothers had hemorrhagic fever symptoms and/or mothers had died within the last 3 days before sampling (full demographic details are given in Supplemental Table S3). Approximately, 79.5% of the 185 samples came from family farms compared with commercial farms. Cattle were identified as either exotic, local, or crossbreed. Of the 157 cows, 65.6% were classified as crossbreed, 25.5% as exotic breeds, and the remainder were classified as local breed. The study was not designed to test for differences in positivity among breeds. According to owners, none of the sampled animals were vaccinated for RVFV. In total, 30.3% (56/185) samples were positive to RVFV—12 goats and 44 cattle. Of those that were positive for RVFV, 26.7% had signs of hemorrhagic disease, whereas 82.1% presented with abortion. All 12 goats that were RVFV positive experienced abortion and one had hemorrhagic fever, whereas 85.0% of cattle experienced abortion (34/40 female cattle) and 14 had hemorrhagic fever. All cases of hemorrhagic disease (*n* = 15) were positive for RVFV via RT-PCR and resulted in death 1–4 days after sample collection, and three of the four were the cases in cows and one case was in a goat.

### Suggested circulation of orthobunyaviruses via RT-PCR.

Among the 129 RVF-negative samples, seven were positive to BUNV and/or BATV. Two samples were exclusively positive to BUNV, two were exclusively positive to BATV, whereas three were positive to all segments of both viruses, which indicates a possible coinfection (Table 1). These seven samples were from the Southern (Nyanza, Ruhango, and Kamonyi districts) or Northern provinces (Gakenke and Gicumbi districts) (Table 1, Supplemental Figure S2). All cases of BUNV and/or BATV suggested infection were in cattle that had suffered abortions.

**Table 1:** Summary of results from RT-PCR and sequencing of amplicons

Sample ID	Species	District	RT-PCR–suggested positivity	BUNV sequencing positivity	BATV sequencing positivity
84	Cow	Gakenke	BUNV+	+	–
120	Cow	Gicumbi	BUNV+	+	–
127	Cow	Gicumbi	BUNV+ and BATV+	+	S
128	Cow	Ruhango	BUNV+ and BATV+	+	S and M
129	Cow	Ruhango	BUNV+ and BATV+	+	S and M
132 (R+)	Cow	Ruhango	BUNV M and NRIV M	M	M*
167 (R+)	Cow	Ngoma	BUNV S and BATV S	S	–
108 (R+)	Cow	Rwamagana	BUNV (M and L), BATV S, and NRIV (S and M)	–	–
198 (R+)	Cow	Kayonza	BATV (S and M)	–	S
144 (R+)	Cow	Kamonyi	BUNV M, BATV S, and NRIV (S and M)	–	M
143	Cow	Kamonyi	BATV+	–	S and M
102 (R+)	Goat	Gatsibo	BATV M and NRIV S	–	–
106 (R+)	Cow	Rwamagana	BUNV M and NRIV M	M	M*
160 (R+)	Cow	Ngoma	BUNV S	–	–
195 (R+)	Cow	Ngoma	BATV S and NRIV S	–	–
109 (R+)	Cow	Rwamagana	BUNV M, BATV S, and NRIV M	–	S† and M*
61	Cow	Nyanza	BUNV S	–	–

BATV = Batai virus; BUNV = Bunyamwera virus; NRIV = Ngari virus; RVFV = Rift Valley fever virus. Amplification results for targeted orthobunyavirus genes and RVFV L segment in sampled animals. Five animals were positive for all three segments of BUNV by sequencing. Others were suggested co-positive for both BUNV and BATV by RT-PCR, but sequencing failed to confirm such in animals 127, 128, and 129. Other animals, including coinfected RVFV+ animals, had evidence of some, but not all three gene segments of BUNV, BATV, and NRIV. This could indicate either the presence of other orthobunyaviruses or novel reassortants co-circulating with RVFV in Rwanda. (+) Indicates positive for all three segments, otherwise individual segments are noted. (R+) Indicates that the sample was RVFV positive via RT-PCR of the L segment.

* Indicates samples that were positive by RT-PCR for NRIV, but sequence BLAST indicated identity with BATV.

† Indicates the one sample where double coverage sampling was not achieved, but BLAST of FWD-only sequence indicates identity to BATV.

Ten RVFV RNA–positive samples had evidence of orthobunyavirus coinfection based on RT-PCR. However, none of them were decisively positive to either BUNV, BATV, or NRIV (Table 1). Overall, two samples amplified the S segment of BUNV, four the M segment, and two the L segment. Three samples in total amplified the BATV S segment and four samples amplified the M segment. There was no amplification of the BATV L segment. Four samples amplified the NRIV S segment, and interestingly none of these samples amplified the BUNV S segment, despite our primers resulting in good cross-amplification. Four samples also amplified the NRIV M segment, and two of those samples also amplified the BATV M segments, whereas the other two did not.

### Confirmation of BUNV circulation and detection of orthobunyavirus gene segments in cattle via sequencing.

All five of the BUNV-positive samples via RT-PCR were also positive via sequencing (Table 1, Supplemental Table S4). Of those that were suggested to be coinfected with BUNV and BATV, all three had at least one segment confirmed by sequencing. In three instances, the M segment was amplified by the NRIV RT-PCR primers and subsequently sequenced using those primers. However, BLAST indicated identity with the BATV M segment. In all cases (106, 109, and 132), neither the RT-PCR nor sequencing suggested some combination of the BUNV_S_BATV_M_BUNV_L_ to prioritize the suggestion of NRIV over BATV. Only three samples had no successful sequencing of amplicon. Accession numbers for sequences are given in Supplemental Table S4.

## DISCUSSION

In this study, we identified RVFV as the causative agent of all cases of hemorrhagic fever among cows and one goat in our sample of these species. Furthermore, RVFV accounted for all clinical disease (hemorrhagic and abortion) in approximately 30% of sampled cases during the largest RVFV outbreak ever documented in Rwanda. The outbreak highly affected the Eastern Province of Rwanda, which was not surprising as it has been shown to be the most suitable region for mosquito life cycles, as this region is characterized by multiple lakes, rice plantations, low altitude (< 2,300 m), low precipitation, and banana plantations in close proximity to households.^60^ Indeed, a mosquito survey conducted in 2011 showed that this region had the highest density of four of the most One Health relevant mosquito species (in the order of highest density to lowest): *Culex* spp., *Aedes* spp., *Coquillettidia* spp., and *Anopheles* spp. *Culex* and *Aedes* are presumed the primary and secondary vectors of RVFV, and *Aedes aegypti* and *Anopheles gambiae* have been shown to be competent vectors for these orthobunyaviruses.^22,61,62^ All districts of the Eastern provinces were affected except Bugesera and Nyagatare districts, where systematic vaccination campaigns had been implemented less than 1 year before the outbreak. This and indication by cattle owners in our study that none of the sampled animals had been vaccinated underscores the utility of high-coverage vaccination programs to preserve the herd health of ruminants to RVFV.

**Figure 1.**
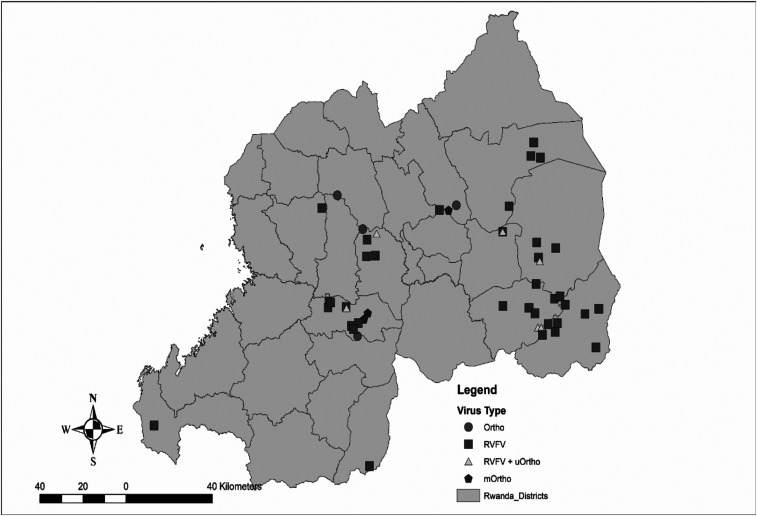
Geographic distribution of RVF+ and suggested orthobunyavirus+ cases: Ortho: Bunyamwera virus (BUNV+) or Batai virus (BATV+) cases, Rift Valley fever virus: RVFV+ cases, RVFV+/uOrtho+: potential coinfection between RVFV and orthobunyaviruses; mOrtho: BUNV+/BATV+ coinfection cases. Suggested positivity is via RT-PCR results.

However, for the first time in Rwanda, sequencing of these samples confirms the presence of BUNV and suggests the presence of BATV and/or NRIV, or perhaps uncharacterized derivatives of these orthobunyaviruses. We showed that these orthobunyaviruses, in a small number of cases, were associated with abortions in a small number of ruminants, similar to RVFV, and therefore contributed to the outbreak of the RVF-like illnesses in Rwanda. Furthermore, these observations support our speculation that these pathogens may be causing illness among cattle in neighboring countries. Batai virus has mainly been detected in Europe and Asia, and primarily in avian species, and this is the second molecular confirmation of BATV in Africa since its original identification in Uganda in 1966.^45,46,48^ This could point to a role for migrating birds in the spread of BATV given the African–Eurasian flyway, and there could be opportunity for its range to be broader than what is currently understood. Figure 1 shows the geographic distribution of RT-PCR RVFV+ cases, orthobunyavirus+ cases, and RVFV+/Ortho+ cases.

Sequencing was, at times, confounded by volume sample, which could account for the discrepancy in the RT-PCR and sequencing results. In several cases, segments were detected and/or sequenced, but the present study was unable to definitively identify these other orthobunyaviruses, again because of resource limitation. However, our data certainly suggest an important future direction to determine whether BUNV, BATV, and/or NRIV co-circulate and can coinfect among one another and with RVFV.

The provenance of NRIV is unknown, but assumed to have occurred because of a coinfection with BUNV and BATV, largely assumed to have happened in the vector.^47^ However, our study suggested that this may occur in cattle, indicating mammals should not be overlooked as vessels of potential reassortant events. In neighboring countries, other orthobunyaviruses related to BUNV circulate, and this raises the question of whether heretofore unidentified reassortants could be occurring in the region.^63,64^

There is a general dearth of knowledge regarding the ecology of arbovirus transmission in Rwanda. Environmental suitability studies on potential vectors and the role of environmental parameters should be conducted to understand transmission dynamics and offer control solutions, including further characterization of these orthobunyaviruses. The scope of this study was the detection of viral RNA among acutely ill animals during an outbreak of RVF-like illness. However, we speculate that seroprevalence will be higher in the general cattle population and perhaps in the human population. Importantly, although RVFV was assumed to be the causative agent of disease in this unprecedented outbreak, there was a high proportion of samples negative for all viruses tested here. The etiology of these cases could be due to any number of pathogens that share clinical manifestation, namely, brucellosis and anaplasmosis. These are important issues warranting further research in the region.

Overall, we have demonstrated that RVFV was responsible for 30% of tested cases with RVF-like illness. We also showed that BUNV and possibly BATV co-circulate with RVFV and were capable of producing acute illness in ruminants. Furthermore, our data suggested that coinfections of orthobunyaviruses and/or RVFV occur and that there may be other orthobunyaviruses circulating in the region that are similarly understudied. Further work is needed to describe the biodiversity of arboviruses in Rwanda, the ecology of such viruses (including vectors, thermal limits of transmission, reservoirs, etc.), and the possibility of causing human disease.

## Supplemental tables, and figures

Supplemental materials
