# Supplementary material for: Identification of Bunyamwera and Possible Other Orthobunyavirus Infections and Disease in Cattle during a Rift Valley Fever Outbreak in Rwanda in 2018
Source: Am J Trop Med Hyg. 2020 Apr 20;103(1):183–9. doi: 10.4269/ajtmh.19-0596 (PMC7356447; doi:10.4269/ajtmh.19-0596)
Supplement: Supplementary file 1 [file tpmd190596.SD1.pdf]

Supplemental Information

Table S1: RT-PCR Primers and sequencing primers

Virus and segment targeted	Primers	Primer sequence	Amplicon size
BUNV-S	Forward primer	5'-CTT CCC AGG ATC AGA GAT GTT-3'	395bp
	Reverse primer	5'-ATT TAG CCC GCT GTC TTT CT-3'	
BUNV- M	Forward primer	5'-GCT TAT GGA TGG GCG TAC AA-3'	651bp
	Reverse primer	5'-GAT GCC TCT GAC CCA GTT AAT-3'	
BUNV- L	Forward primer	5'-AAT CCA GAG GCC CTA GGT ATA A-3'	767bp
	Reverse primer	5'-GAC CAA GGC TCT TGC TCT ATC- 3'	
BATV-S	Forward primer	5'-GAC CCA GAG GTT GCA TAC ATT A-3'	394bp
	Reverse primer	5'-CAG ACC CTG GAA AGA ATG AGA G-3'	
BATV- M	Forward primer	5'-GAA CTA ATC CCT GCC TCA CAA G-3'	847bp
	Reverse primer	5'-GAC ACT CTC CTC AAC TGC TTT-3'	
BATV-L	Forward primer	5'- TGT ATC ATC ACC CGG CTT ATT C-3'	798bp
	Reverse primer	5'-CAG AGG GTT TGA CAG CCT ATA TT-3'	
NRIV-S	Forward primer	5'-TGC TAA CAC CAG TAC TTT-3'	504bp
	Reverse primer	5'-ACC TCT GTC GCA TTG TCT TT- 3'	
NRIV-M	Forward primer	5'-TGG GTG CCT TGC TGT AAA TA - 3'	914bp
	Reverse primer	5'-CAC TGA TCC CTT CAT CCC TAA C- 3'	
NRIV-L	Forward primer	5'-AGC CAG CAC TAC AAG ATT AGA- 3'	785bp
	Reverse primer	5'-TCT CCT TGC TCA TCA CCA TTA- 3'	

Supplemental Table S2: Confirmation of primer specificity for sequencing, validation using viral socks from WHO reference laboratory. The % identity was confirmed using NCIB BlastNT and the accession number of the top hit given.

Virus Stock	Segment	% identity	Accession
BATV	S	100	X73464
	M	99.77	JX846596
	L	99.86	JX846597
BUNV	S	95.59	D00353
	M	97.34	MY091919
	L	99.71	X14383

NRIV	S	99.77	JX857316
	M	99.75	KC608153
	L	100	KC608152

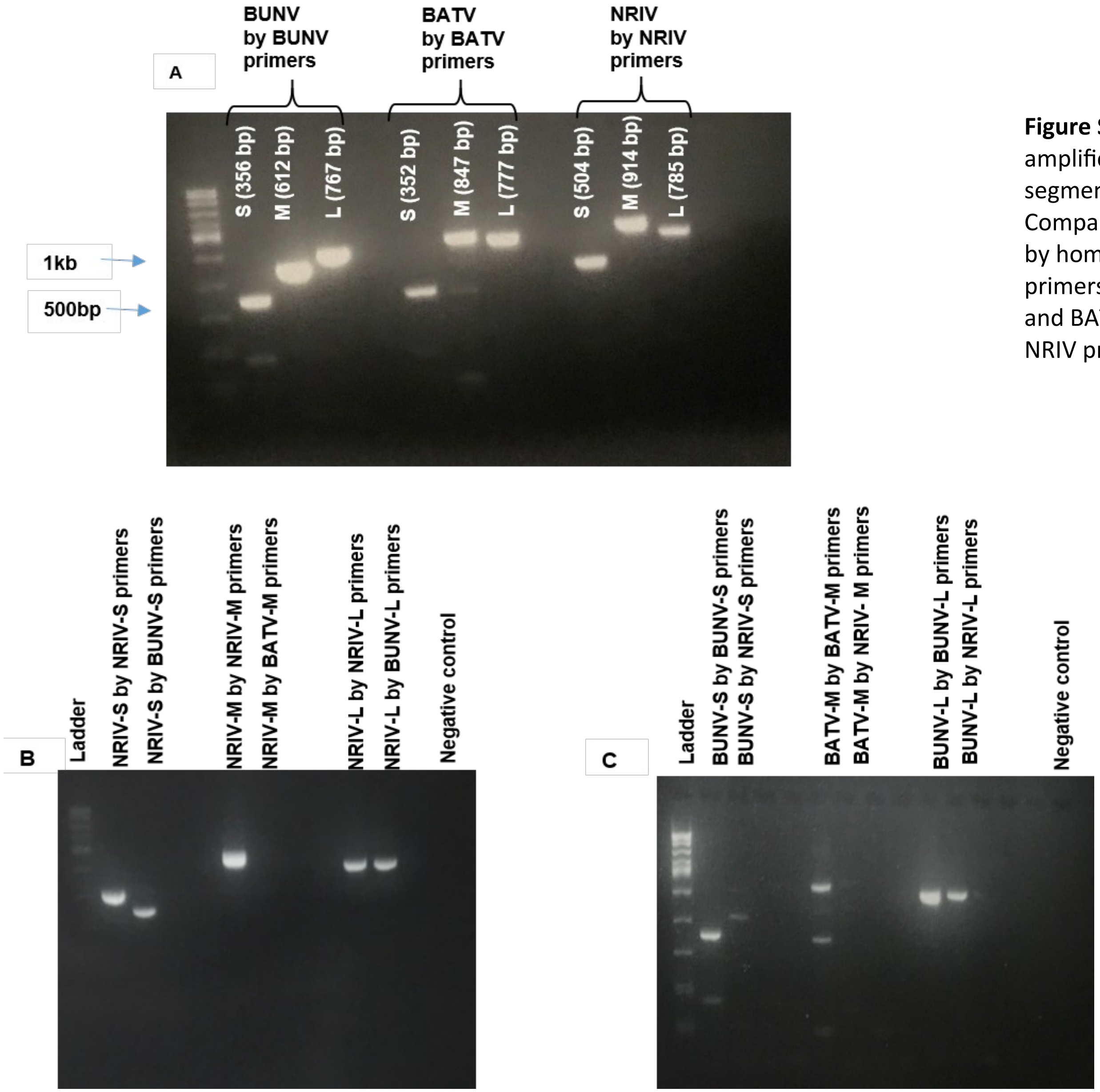

Figure S1: 1% agarose gels showing amplification and cross-amplification of PCR primers to BUNV, BATV, and NRIV gene segments. **A.** Amplification by homologous primers. **B.** Comparison between amplification of NRIV genome segments by homologous primers and its parental genome segment primers, **C.** Comparison between amplification of BUNV (S, L) and BATV (M) genome segments by homologous primers and by NRIV primers.

Supplemental Figure S2: Visualization on 2% agarose gel of DNA bands for amplified regions of BUNV and BATV S, M, L segments of these 7 samples.

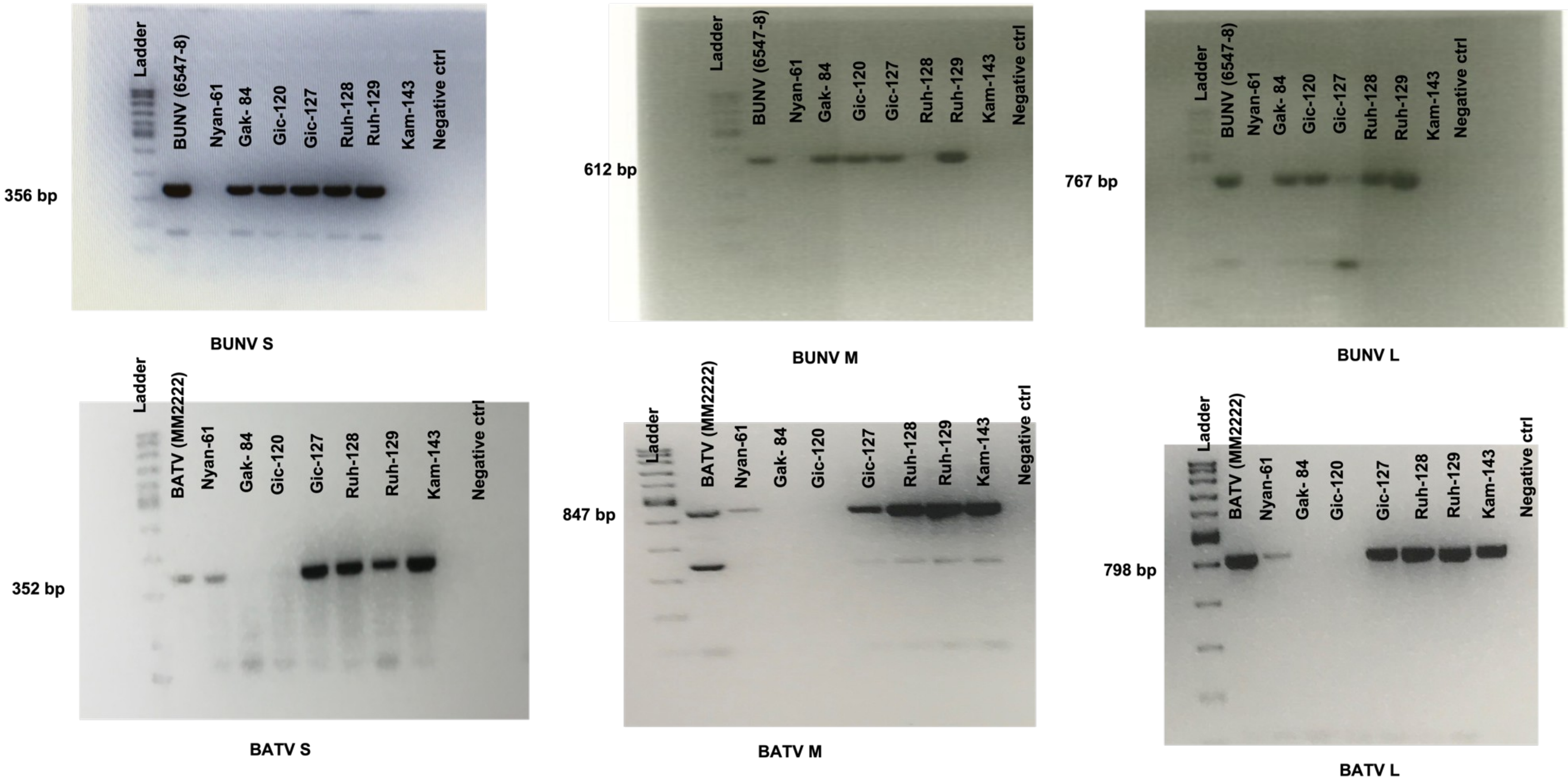

Table S3: Demographics of the sample population.

Samples categories			Total number of samples collected	RVF positive samples (% positive of demographic category)
Overall prevalence			185	56 (30.27%)
Species		Cattle	157	44 (28.02%)
		Goats	28	12 (48.85%)
Sex		Female	181	55 (30.38%)
		Male	4	1 (25%)
Type of farm		Family	147	41 (27.89%)
		Commercial	38	15 (39.47%)
Breeds (cattle)		Local	16	6 (37.5%)
		Crossed	102	30 (29.41%)
		Exotic	39	8 (20.51%)
Clinical symptoms		Abortion	157	46 (29.29%)
		Hemorrhagic fever	15	15 (100%)
Geographic distribution	Eastern province	Ngoma	41	10 (24.39%)
		Kirehe	32	7 (21.87%)
		Kayonza	18	6 (33.33%)
		Gatsibo	28	12 (42.85%)
		Rwamagana	5	4 (4/5)
	Southern province	Ruhango	17	9 (52.94%)
		Kamonyi	15	4 (26.66%)
		Muhanga	3	-
		Nyanza	5	-
		Gisagara	1	1 (1/1)
	Northern province	Gakenke	5	-
		Gicumbi	10	-
	Western province	Ngororero	1	1 (1/1)
		Rusizi	1	1 (1/1)
	Kigali city	Gasabo	3	1 (1/3)

Table S4: BLAST results for sequenced segments of positive samples.

Virus	Sample ID	Segment primer	GenBank Accession #
BUNV	84	S	MT043345
		M	MT022496
		L	MT022495
	120	S	MT043346
		M	MT022499
		L	MT022498
	127	S	MT043347
		M	MT022501
		L	MT022500
	129	S	MT022506
		M	MT022505
		L	MT022504
	128	S	MT043348
		M	MT022503
		L	MT022502
	106	M	MT022497
	132	M	MT022507
	167	S	MT043349
BATV	198	S	MT022516
	144	M	MT022515
	132	M	MT022493
	127	S	MT022508
	109	S*	N/A
		M	MT022494
	143	S	MT022514
		M	MT022513
	128	S	MT022510
		M	MT022509
	106	M	MT022492
	129	S	MT022512
		M	MT022511

*Only FWD sequence was able to be obtained due to sample volume depletion. BLAST top hit JX846595.1 with 99.68% identity. FWD sequence available upon request.
